# Supplementary material for: Diagnostic Value of Single-Photon Emission Computed Tomography/Computed Tomography Scans with Tc-99m HDP in Cervicogenic Headache
Source: J Clin Med. 2020 Feb 2;9(2):399. doi: 10.3390/jcm9020399 (PMC7074323; doi:10.3390/jcm9020399)
Supplement: Supplementary file 1 [file jcm-09-00399-s001.docx]

[Supplement]

**Table S1.** The observed number of patients who meet the loose criteria of successful response at 6 months follow-up between each group.

|  | Success  (*n* = 13) | Non-success  (*n* = 10) | *p*-value |
| --- | --- | --- | --- |
| SPECT/CT group | 7 (53.85) | 4 (40.00) | 0.532 |
| Control group | 6 (46.15) | 6 (60.00) |  |
| SPECT/CT (+) group | 7 (100.00) | 1 (25.00) | 0.003 |
| SPECT/CT (-) group | 0 (0.00) | 3 (75.00) |  |
| SPECT/CT (+) group | 7 (53.85) | 1 (14.29) | 0.094 |
| Control group | 6 (46.15) | 6 (85.71) |  |

The loose criteria of successful response were defined as any of the followings was satisfied: > 50% (or 4-point) reduction from baseline in the neck and occipital VAS score, ≥ 30% decrease from baseline in the NDI, and ≤ 2 points on the GPE scale. SPECT/CT, single-photon emission computed tomography/computed tomography; VAS, visual analog scale; NDI, neck disability index; GPE, global perceived effect.
